# Functional recovery of demineralized dentin using a glutamic acid-modified electrospun scaffold: A multimodal in vitro characterization

**DOI:** 10.1016/j.jobcr.2025.11.005

**Published:** 2025-11-14

**Authors:** Aruna Krishnan, Sandhya Raghu, Govindaraj Perumal, Jayalakshmi Somasundaram, Nishitha Arun

**Affiliations:** a Department of Conservative Dentistry and Endodontics, Saveetha Dental College and Hospitals, Saveetha Institute of Medical and Technical Sciences, Saveetha University, Chennai, India; b Department of Biomedical Engineering, School of Dental Medicine, University of Connecticut (UConn) Health, Farmington, CT, USA

## Abstract

**Background:**

Dentin remineralization poses a significant challenge in restorative dentistry due to the complex structure and mineral-organic composition of dentin. Electrospun nanofibrous scaffolds present a biomimetic platform to facilitate mineral deposition. Functionalization of the scaffold, achieved through the incorporation of biomolecules like glutamic acid, which mimics non-collagenous proteins, may improve remineralization.

**Objective:**

To assess the remineralization potential of a glutamic acid-loaded polycaprolactone/nanohydroxyapatite (PCL/nHA/Glu) scaffold on acid-demineralized dentin, through surface and mechanical characterization.

**Methods:**

Nanofibrous scaffolds of PCL/nHA and PCL/nHA/Glu were fabricated and applied to demineralized human dentin discs. The samples were immersed in simulated body fluid for 7, 14, and 28 days. Changes in morphology and composition were analyzed using scanning electron microscopy (SEM) and energy-dispersive X-ray spectroscopy (EDS). Surface topography was assessed by atomic force microscopy (AFM), and mechanical properties were evaluated via nanoindentation, with statistical analysis conducted using one-way ANOVA.

**Results:**

SEM imaging showed progressive mineral deposition in both scaffold groups, with the PCL/nHA/Glu group demonstrating organized crystallite formation and almost complete tubule occlusion by Day 28. EDS indicated earlier and higher Ca and P incorporation in the PCL/nHA/Glu group. AFM showed significant reductions in surface roughness later, while nanoindentation revealed increased elastic modulus and hardness in the PCL/nHA/Glu group by Day 14, indicating mechanical recovery.

**Conclusion:**

Glutamic acid-functionalized scaffolds significantly enhanced the remineralization of demineralized dentin, promoting organized mineral deposition and restoration of mechanical properties. These findings support using amino acid-modified scaffolds in dentin tissue engineering.

**Clinical significance:**

Functionalization with amino acids is crucial for mimicking the activity of non-collagenous proteins, transforming passive ion delivery systems into bioactive platforms for dentin regeneration. These scaffolds could serve as alternatives to traditional materials in restorative dentistry, aiding in the treatment of dental caries, dentin hypersensitivity, and enhancing pulp vitality and regeneration.

## Highlights

• Observed very good remineralization of demineralized dentin.
• Bead-free, randomly aligned nanofibers with uniform surface.
• Improved nanofiber diameter.
• Controlled mineralization of Glu-loaded nanofibers in a 3D manner.
• Biomimetic apatite-like mineralization with Ca-P ratio.

## Graphical abstract

**Figure:**
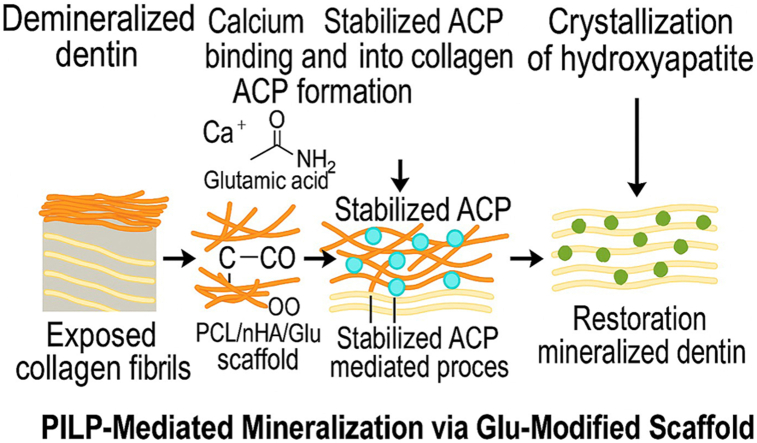

## 1 Introduction

Dentin, a structurally complex mineralized tissue, plays a key role in maintaining tooth integrity. It is composed mainly of type I collagen fibrils integrated with carbonated apatite nanocrystals, forming a composite structure with hierarchical organization.^1^ Dental caries initially leads to demineralization of the inorganic portion, leading to reduced mechanical strength. It increases the vulnerability to enzymatic degradation of the organic portion.^2^^,^^3^ Dentin remineralization is necessary to reverse the effects of the enzymatic degradation of the collagen matrix. Conventional top-down approaches often fail to regenerate the native nanostructure, producing only superficial mineral layers with limited mechanical restoration.^4^^,^^5^ An emerging paradigm in dental tissue engineering is biomimetic remineralization, which aims to restore the physiological deposition of mineral phases within the collagen fibrils. This process depends on analogues of non-collagenous proteins (NCPs), such as dentin matrix protein 1 (DMP1) and dentin phosphophoryn (DPP), to stabilize amorphous calcium phosphate (ACP) precursors and guide their infiltration into collagen via the polymer-induced liquid-precursor (PILP) mechanism.^6^^,^^7^ The intrafibrillar transformation of ACP into hydroxyapatite is essential for restoring dentin's biomechanical function.^8^

**Fig. 1:**
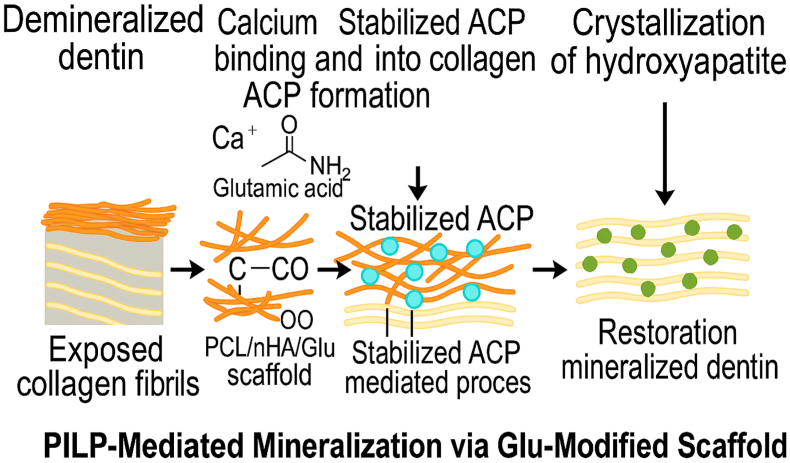
Schematic illustration of the proposed mechanism of dentin remineralization via a PCL/nHA/Glu nanofibrous scaffold. The negatively charged carboxyl groups of Glu acid interact with calcium ions to stabilize ACP, which subsequently infiltrates the collagen matrix. This stabilized ACP undergoes intrafibrillar crystallization into hydroxyapatite, thereby restoring the mineralized dentin structure via a PILP pathway.

Nanofibrous scaffolds have been employed to replicate the extracellular matrix (ECM) microenvironment that facilitates this biological process. Nanofibrous scaffolds are commonly fabricated by the electrospinning process. It possesses high surface area, porosity, and morphological resemblance to native ECM, which facilitates improved cell adhesion, proliferation, and mineral nucleation.^9^ Polycaprolactone (PCL) is a widely used polymer for nanofibrous scaffold fabrication. It demonstrates controlled biodegradation aligning with tissue mineralization, ease of processing, and FDA approval for biomedical applications.^10^ But the intrinsic hydrophobicity of PCL restricts protein adsorption and cell–scaffold interactions, which limits its regenerative potential.^11^^,^^12^ To improve biofunctionality, PCL scaffolds are often modified with nanohydroxyapatite (nHA), a biomimetic calcium phosphate material that is similar to the inorganic component of dentin and bone.^13^ nHA increases mechanical strength and surface bioactivity by releasing calcium and phosphate ions. It also has the capacity to enhance odontogenic differentiation and improve cell attachment through increased scaffold wettability and surface roughness.^14^, ^15^, ^16^ The brittleness of nHA is reduced when integrated into PCL matrices, creating composites for use in functional tissue repair. Acidic amino acids such as glutamic acid (Glu) are incorporated into the scaffolds due to their functional similarity to mineralization domains found in NCPs. DMP1 and DPP contain Glu-rich sequences that act as nucleation sites by binding calcium ions, stabilizing ACP phases, and accelerating crystallization into apatite.^17^ L-Glu has been shown to promote dentin remineralization by simulating the electrostatic interactions of NCPs with mineral ions.^18^ Poly-γ-glutamic acid (PGA), composed of Glu residues, also exhibits strong nucleation capacity. In addition, PGA can serve as a bridge between collagen and HA due to its high content of carboxyl groups in its side chains.^19^ Based on this concept, we previously prepared and characterized a Glu acid-loaded nanofibrous scaffold comprising PCL and nHA by electrospinning.^20^ The PCL/nHA/Glu scaffold exhibited uniform, bead-free fibers, improved hydrophilicity, controlled Glu release, and excellent biomineralization based on SEM-EDS and simulated body fluid studies. The scaffold also has high cytocompatibility and cell adhesion with human dental pulp stem cells (hDPSCs), suggesting a favorable microenvironment for dentin mineralization. FTIR analysis confirmed the successful incorporation of glutamic acid. XRD analysis confirmed the formation of crystalline and amorphous hydroxyapatite phases, which collectively contributed to the scaffold's enhanced surface bioactivity. As shown in Fig. 1, this PILP-mediated mechanism involves negatively charged Glu residues attracting positively charged calcium ions. It stabilizes ACP nano precursors and facilitates their infiltration into demineralized collagen fibrils. Subsequently, crystal nucleation and growth of hydroxyapatite happen, restoring the structural and mechanical integrity of dentin. While previous studies have employed amino acid-functionalized materials or PILP-based strategies for dentin remineralization, the present study aims to comprehensively evaluate a glutamic acid-loaded electrospun PCL/nHA scaffold using multimodal surface and mechanical characterization.

Based on this rationale, the present study aims to explore the remineralization potential of PCL/nHA/Glu nanofibrous scaffold on demineralized dentin disc. To evaluate the remineralization efficacy of a PCL/nHA/Glu nanofibrous scaffold, characterization techniques such as scanning electron microscopy (SEM), energy-dispersive X-ray spectroscopy (EDS), atomic force microscopy (AFM), and nanoindentation were used. The objective of our study is: (i) to assess scaffold-guided remineralization of demineralized dentin by immersion in simulated body fluid (SBF), (ii) To analyze the surface morphology and elemental composition of remineralized dentin using SEM and EDS, (iii) To characterize nanoscale surface topography using AFM. (iv) To evaluate mechanical recovery of dentin via nanoindentation-based measurements of reduced elastic modulus and hardness, and (iv) To statistically compare the remineralization efficacy between PCL/nHA and PCL/nHA/Glu scaffolds.

## 2 Materials and methods

### 2.1 Dentin sample preparation and demineralization

Extracted human third molars, free from caries, cracks, or restorations, were collected in accordance with institutional ethical guidelines. The study protocol was approved by the Institutional Review Board of Saveetha Institute of Medical and Technical Sciences, Chennai, India (Approval No. SRB/SDC/ENDO-2201/23/TH-168). Following extraction, specimens were stored in 0.1 % thymol solution at 4 °C to prevent microbial contamination. Mid-coronal dentin discs (∼1 mm thick) were sectioned using a water-cooled, low-speed diamond saw (Isomet, Buehler, USA). Exposed surfaces were standardized through sequential polishing with 600-, 800-, and 1200-grit silicon carbide abrasive papers under constant water irrigation. Demineralization was achieved by applying 37 % phosphoric acid to the dentin surface for 2 min at room temperature, followed by thorough rinsing with deionized water. Demineralized specimens were then stored in phosphate-buffered saline (PBS, pH 7.4) at 4 °C, following the methodology described by Liang et al.^21^ Scaffold fabrication and initial physicochemical characterization (SEM, EDS, FTIR, XRD, and contact angle analysis) were conducted as previously reported^20^ and are not detailed here.

### 2.2 Remineralization protocol

**Fig. 2:**
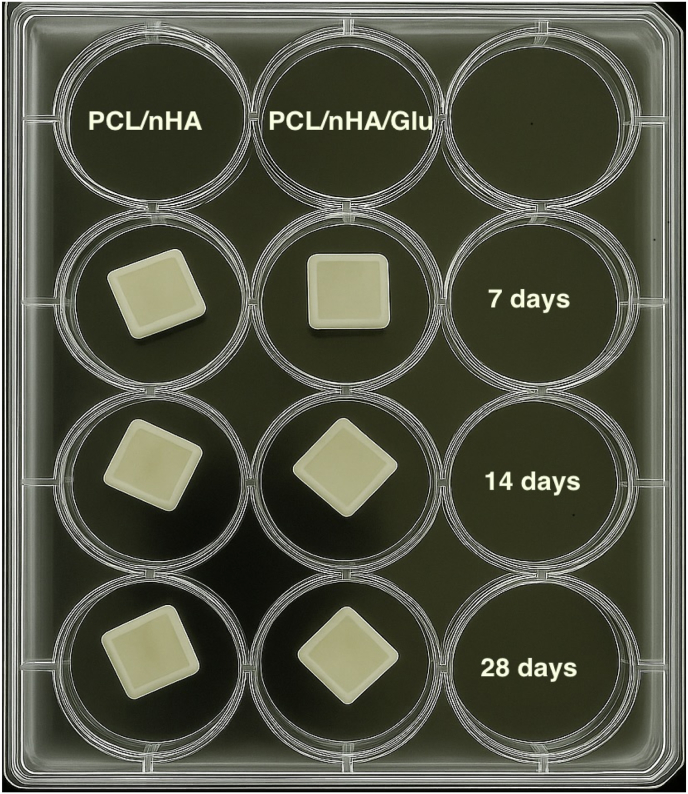
Schematic representation of the in vitro remineralization setup. Demineralized dentin discs were placed in direct contact with either PCL/nHA or PCL/nHA/Glu electrospun scaffolds, immersed in simulated body fluid (SBF), and incubated at 37 °C for 7, 14, and 28 days to evaluate scaffold-mediated mineralization.

Our previously reported study with glutamic acid and nHA incorporated nanofibrous scaffolds (PCL/nHA/Glu) demonstrated enhanced mineralization and improved cellular functionalities of human dental pulp stem cells (hDPSCs) compared to PCL/nHA.^20^ In this study, we utilized scaffolds for demineralized dentin specimens to evaluate their remineralization capacity. The demineralized dentin samples were randomly assigned to one of the following groups: **Group 1:** Demineralized dentin only (negative control), **Group 2:** PCL/nHA scaffold, and **Group 3:** PCL/nHA/Glu scaffold. Each dentin disc was placed in direct contact with its designated scaffold and positioned within a sterile 12-well tissue culture plate. Simulated body fluid (SBF, pH 7.4; 2 mL) was added to each well. The plates were incubated at 37 °C for 7, 14, and 28 days as shown in Fig. 2. The SBF medium was replaced every 48 h to maintain ionic equilibrium. At each time point, specimens were rinsed with deionized water to remove loosely bound minerals and dried in a vacuum desiccator for 24 h. This setup was designed to mimic physiological remineralization environments and allow assessment of scaffold-mediated mineral deposition over time.

### 2.3 SEM–EDS

Gold-sputtered specimens were examined using field emission scanning electron microscopy (FE-SEM; JSM IT800, JEOL Ltd., Tokyo, Japan) at × 1000 and × 5000 magnifications. Surface changes were documented. EDS integrated with SEM was employed to analyze elemental distribution. Elemental spectra were collected in triplicate for each sample group. Elemental weight percentages of calcium and phosphorus were obtained, and Ca/P molar ratios were computed to evaluate the quality of remineralization.

### 2.4 AFM analysis

Surface nanostructure was evaluated using AFM (Nanosurf C3000, Switzerland) in tapping mode with a silicon nitride cantilever (Tap190Al-G, tip radius <10 nm). Scans of 10 μm × 10 μm at 256 lines/frame resolution were captured. Surface roughness parameters included: (i) Average roughness (Ra), (ii) Root mean square roughness (Rq), (iii) Maximum peak-to-valley height (Ry) were analyzed using proprietary software. Each sample was scanned in triplicate at 7, 14, and 28 days post-treatment following the protocol of Bertassoni et al.^22^ All scans were performed under ambient conditions. Measurements were averaged across three independent regions per sample.

### 2.5 Nanoindentation for mechanical evaluation

Nanoindentation was performed using a UNHT nanoindenter (Anton Paar, Austria) with a Berkovich diamond tip (serial BBF-03). Indentations targeted intertubular dentin regions to avoid variability associated with tubule orientation and content. Test parameters: Maximum load: 5000 μN; Loading/unloading rate: 10,000 μN/min; Dwell time: 10 s; and Approach/retract speed: 2000 nm/min. Measured outputs: Reduced Elastic Modulus (Er); and Hardness (HIT). Mechanical parameters were derived using the Oliver and Pharr method, assuming a Poisson's ratio of 0.30 for dentin. Tests were conducted under dry conditions to eliminate hydration-induced tip drift and ensure reproducibility.

### 2.6 Statistical analysis

Statistical analysis was conducted using IBM SPSS Statistics (Version 26.0). Er and HIT values were expressed as mean ± standard deviation (SD) for each group (n = 3). One-way ANOVA was used to compare groups, followed by Tukey's HSD post hoc test for pairwise analysis. Statistical significance was set at p < 0.05.

## 3 Results

### 3.1 Surface morphology analysis by SEM

**Fig. 3:**
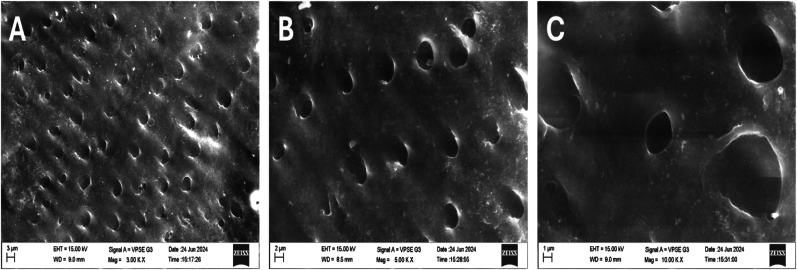
**Control:** SEM image of demineralized dentin before remineralization showing open dentinal tubules and a rough, mineral-depleted surface morphology at 3 different magnifications. This served as the **negative control**, providing a baseline against which scaffold-mediated remineralization effects were evaluated.

Baseline SEM analysis of the acid-etched dentin (Fig. 3) demonstrated pronounced surface roughness, widened dentinal tubules, and a depleted peritubular matrix—hallmarks of successful demineralization. Demineralized dentin was included as a negative control to establish the baseline mechanical and structural condition prior to scaffold-mediated remineralization. The intertubular matrix appeared depleted, with minimal surface structure remaining, indicative of successful demineralization.

**Fig. 4:**
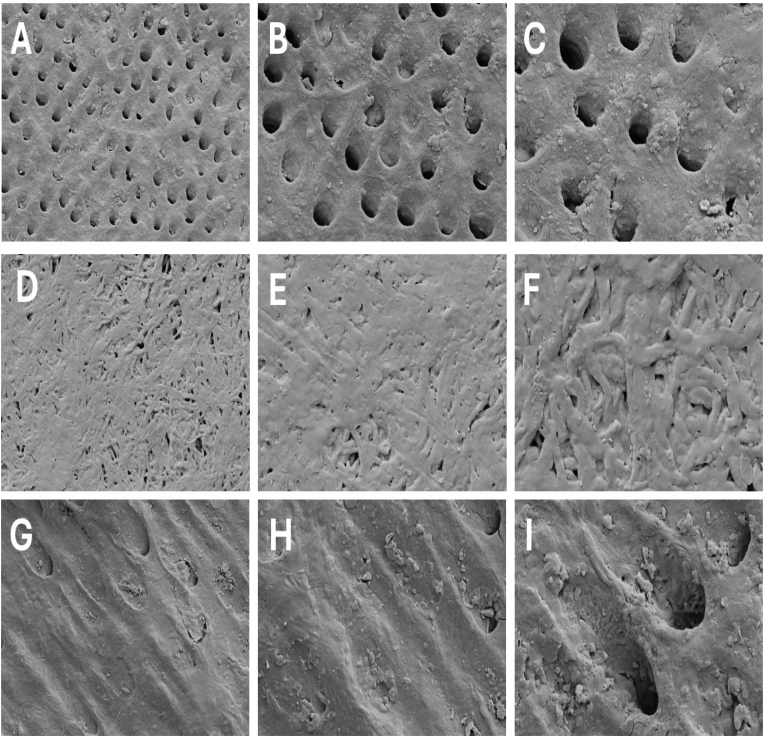
SEM micrographs showing remineralization of demineralized dentin treated with PCL/nHA scaffold over 7, 14, and 28 days. (A–C): 7 days (magnifications × 5000, × 10,000, × 15,000); (D–F): 14 days; (G–I): 28 days. Gradual mineral deposition and progressive tubule occlusion were evident across time, reflecting scaffold-induced hydroxyapatite nucleation and growth.

**Fig. 5:**
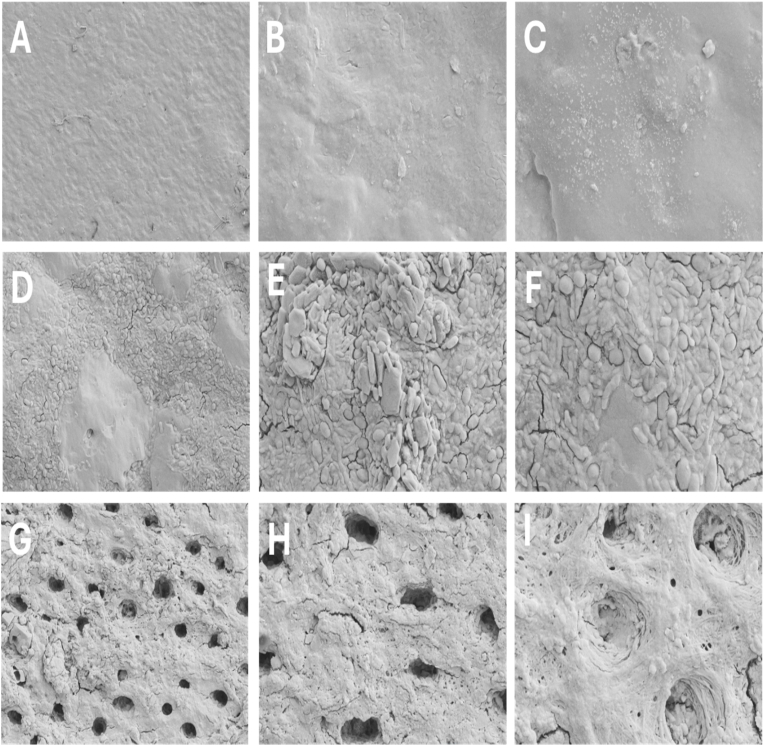
SEM micrographs illustrating remineralization of demineralized dentin treated with PCL/nHA/Glu scaffold over 7, 14, and 28 days. (A–C): 7 days (magnifications × 5000, × 10,000, × 15,000); (D–F): 14 days; (G–I): 28 days.

Subsequent SEM imaging evaluated the surface remineralization dynamics in both scaffold-treated groups at 7, 14, and 28 days (Fig. 4, Fig. 5). In the **PCL/nHA group** (Fig. 4), at Day 7, early-stage mineralization was observed in the form of scattered apatite-like particulates localizing around dentinal tubules, with limited intertubular coverage suggesting nucleation initiation predominantly at exposed tubule walls. By Day 14, these deposits appeared more clustered, though still sparse and morphologically irregular. At Day 28, partial tubule occlusion was evident with globular formations, indicating moderate but incomplete scaffold-guided mineral integration.

Time-dependent increase in mineral accumulation, organized crystal morphology, and extensive tubule occlusion demonstrates enhanced biomimetic potential conferred by glutamic acid functionalization. In contrast, the **PCL/nHA/Glu group** (Fig. 5) exhibited accelerated and spatially uniform remineralization. By Day 7, granular crystal assemblies partially occluded dentinal tubules. At day 14, densely packed globular aggregates extended over both peritubular and intertubular regions, forming a contiguous mineral layer. By day 28, the scaffold facilitated nearly complete tubule occlusion and organized mineral bridging, mimicking native dentin architecture. The glutamic acid functionalized scaffold promoted earlier and more spatially uniform mineral deposition than PCL/nHA, with progressive transformation from scattered crystallites to a contiguous mineral layer. This pattern suggests enhanced nucleation and maturation consistent with calcium carboxylate mediated mineral growth.

### 3.2 EDS analysis

**Fig. 6:**
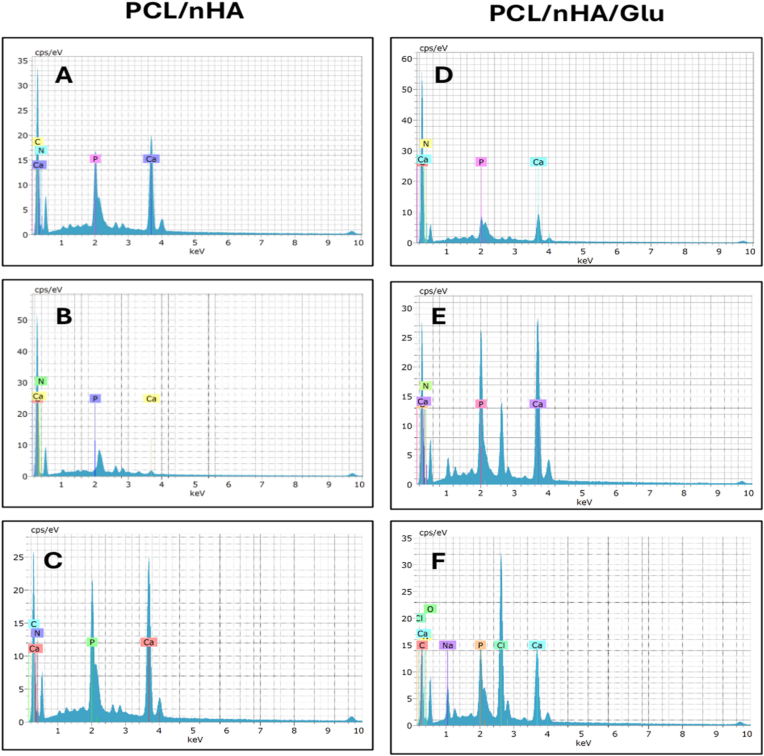
EDS spectra of remineralized dentin surfaces at 7, 14, and 28 days. (A–C): PCL/nHA group; (D–F): PCL/nHA/Glu group. Elemental peaks corresponding to calcium (Ca), phosphorus (P), oxygen (O), nitrogen (N), carbon (C), sodium (Na), and chlorine (Cl) were identified. The PCL/nHA/Glu group exhibited earlier onset and higher intensity of Ca and P peaks, along with a distinct N signal, indicating enhanced and scaffold-guided hydroxyapatite-like mineral formation.

EDS was performed to evaluate the elemental composition and progression of mineral deposition on the dentin surface over time (Fig. 6). The control PCL/nHA group exhibited a gradual increase in calcium (Ca) and phosphorus (P) peak intensities from Day 7 to Day 28, indicating ongoing but limited mineral accumulation. Early spectra (Day 7) showed minor nitrogen (N) and carbon (C) signals, likely reflecting residual scaffold-organic interactions, but Ca and P peaks remained low, consistent with sparse mineral nucleation. By contrast, the PCL/nHA/Glu group demonstrated accelerated and intensified mineralization. EDS confirmed accelerated mineralization in the PCL/nHA/Glu group, reflected by early and intense Ca and P signals and the presence of nitrogen derived from glutamic acid. The reduced Ca/P ratio over time indicates maturation of hydroxyapatite-like phases and improved mineral stoichiometry. These findings corroborate the SEM results and suggest that glutamic acid enhances scaffold bioactivity by mimicking non-collagenous protein domains involved in biomineralization.

### 3.3 Surface topography analysis via AFM

**Fig. 7:**
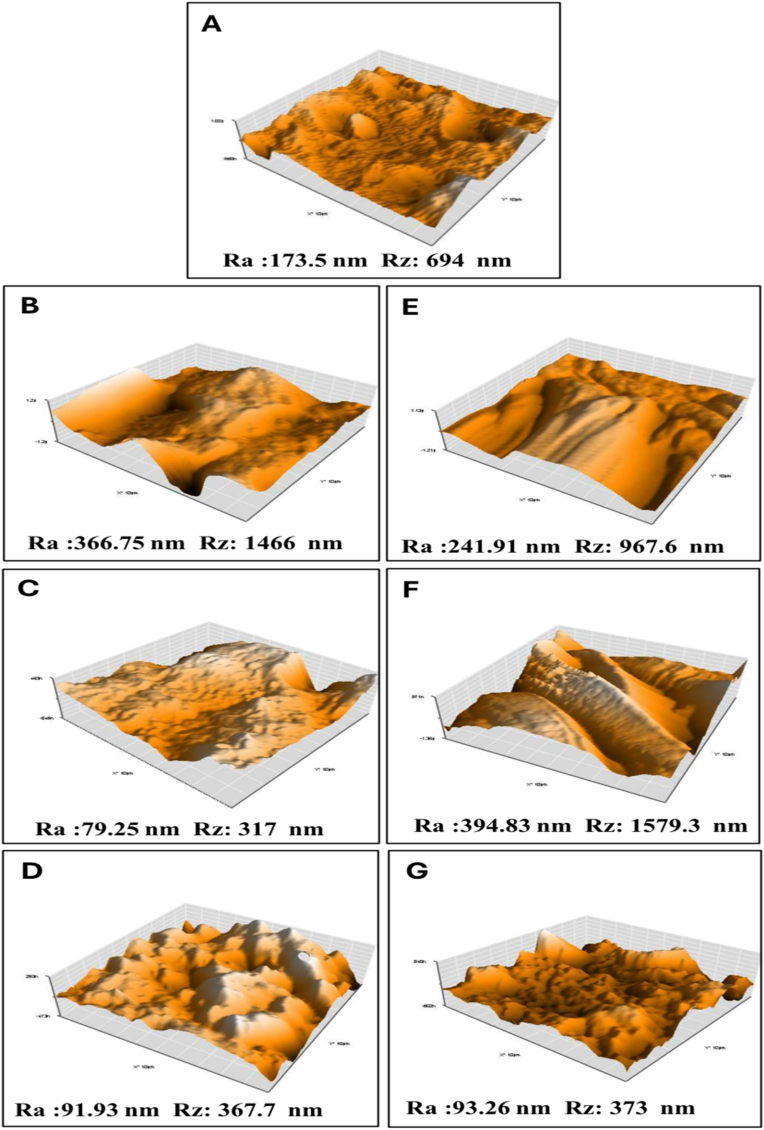
Representative 3D AFM topography images (10 μm × 10 μm) of dentin surfaces: (A) demineralized dentin; (B–D) PCL/nHA at 7, 14, and 28 days; (E–G) PCL/nHA/Glu at 7, 14, and 28 days. Corresponding roughness parameters are annotated for each image.

AFM was employed to characterize the nanoscale topography of demineralized dentin surfaces before and after scaffold-mediated remineralization. Quantitative roughness parameters—average roughness (Ra), root mean square roughness (Rq), maximum peak-to-valley height (Ry), and calculated Rz (≈4 × Ra)—were used to evaluate surface architecture over 7, 14, and 28 days. Results are summarized in Table 1, and representative 3D topography images are presented in Fig. 7. The demineralized dentin group exhibited a high roughness value indicating fibrillar collapse and mineral loss. Both scaffolds promoted surface reorganization over time; however, the PCL/nHA/Glu scaffold showed a more dynamic pattern characterized by early globular mineral aggregation followed by surface homogenization at 28 days. **This reduction in surface roughness corresponds to the transformation of amorphous calcium phosphate into a compact, organized mineral layer, indicating a controlled maturation process guided by glutamic acid functionalization.** Overall, AFM roughness parameters reflected progressive topographical recovery, most pronounced in the PCL/nHA/Glu group.

**Table 1:** Surface roughness parameters (Ra, Rq, Ry, Rz) of dentin samples obtained by AFM analysis. Values reflect scaffold-mediated changes in nanoscale architecture at 7, 14, and 28 days.

Group	Ra (nm)	Rq (nm)	Ry (nm)	Rz (nm) (≈4 × Ra)
**Demineralized dentin**	173.50	199.69	816.54	694.00
**PCL/nHA – 7 days**	366.65	465.06	2071.40	1466.60
**PCL/nHA – 14 days**	79.25	98.47	438.68	317.00
**PCL/nHA – 28 days**	91.93	108.65	385.60	367.70
**PCL/nHA/Glu –** **7 days**	241.91	273.88	1044.40	967.60
**PCL/nHA/Glu –** **14 days**	394.83	477.37	2105.30	1579.30
**PCL/nHA/Glu –** **28 days**	93.26	116.05	560.04	373.00

Ra = average roughness; Rq = root mean square roughness; Ry = maximum peak-to-valley height; Rz = approximated as 4 × Ra.

### 3.4 Mechanical properties of remineralized dentin via nanoindentation

**Fig. 8:**
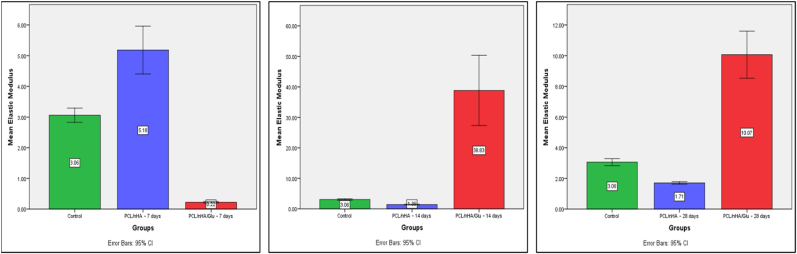
Bar graph showing mean Reduced Elastic Modulus (Er) values (±SD) at 7, 14, and 28 days. The PCL/nHA/Glu group showed significantly higher values at 14 and 28 days (*p* < 0.001).

**Fig. 9:**
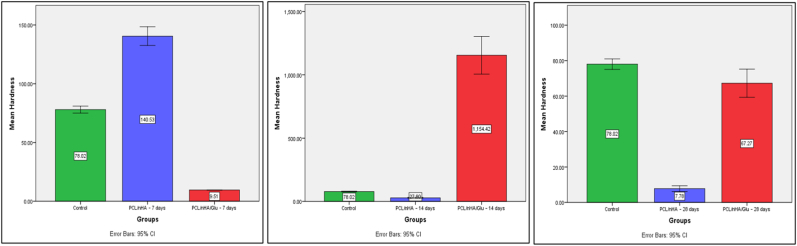
Bar graph showing mean Hardness (HIT) values (±SD) at 7, 14, and 28 days. A significant enhancement in the PCL/nHA/Glu group was observed at Day 14 (*p* < 0.001), with sustained enhancement at Day 28.

Nanoindentation was employed to quantify the mechanical recovery of demineralized dentin following scaffold-mediated remineralization. Both Er and HIT were evaluated using a Berkovich diamond tip under dry conditions. Complete data are shown in Table 2, and statistical comparisons are illustrated in Fig. 8, Fig. 9.

**Table 2:** Mechanical properties of dentin after scaffold-mediated remineralization (mean ± SD, *n* = 3).

Group	Er (GPa)	HIT (MPa)
**Demineralized dentin**	3.06 ± 0.09	78.02 ± 1.19
**PCL/nHA – 7 days**	5.18 ± 0.31	140.53 ± 3.21
**PCL/nHA – 14 days**	1.39 ± 0.04	27.80 ± 1.89
**PCL/nHA – 28 days**	1.71 ± 0.03	7.77 ± 0.67
**PCL/nHA/Glu – 7 days**	0.22 ± 0.01	9.51 ± 0.05
**PCL/nHA/Glu – 14 days**	38.83 ± 4.63	1154.42 ± 59.96
**PCL/nHA/Glu – 28 days**	10.07 ± 0.61	67.27 ± 3.18

Er = Reduced Elastic Modulus; HIT = Hardness.

#### 3.4.1 Er

Demineralized dentin exhibited the lowest modulus reflecting structural degradation due to mineral loss. At 7 days, the **PCL/nHA group** showed a significant increase in Er, which was significantly higher than the **PCL/nHA/Glu group** (*p* < 0.001). However, by 14 days, the trend reversed: **PCL/nHA/Glu** demonstrated a significant increase in modulus, significantly exceeding both **PCL/nHA** and the control (*p* < 0.001). At 28 days, **PCL/nHA/Glu** maintained a significantly higher modulus compared to **PCL/nHA** group (*p* < 0.001), though the value had declined relative to Day 14. This decline in mechanical properties at Day 28 in the scaffold may reflect **restructuring of early-formed mineral**, possible **scaffold degradation**, or **ion depletion**, consistent with dynamic remodeling processes. **One-way ANOVA** confirmed statistically significant differences across groups at each time point (*p* < 0.001). **Tukey's HSD post hoc test** showed that at Day 14 and Day 28, the **PCL/nHA/Glu group** was significantly different from both **PCL/nHA** and the control (*p* < 0.001), affirming its superior capacity for mechanical reinforcement.

#### 3.4.2 HIT

At Day 7, **PCL/nHA** showed the highest hardness, significantly greater than both the control and **PCL/nHA/Glu** (*p* < 0.001). However, at Day 14, **PCL/nHA/Glu** demonstrated a dramatic increase in hardness significantly higher than both **PCL/nHA** and the control (*p* < 0.001). By Day 28, **PCL/nHA/Glu** retained significantly higher hardness compared to **PCL/nHA** (*p* < 0.001), although reduced from Day 14 levels. These results indicate a delayed yet robust mineralization response in the **PCL/nHA/Glu** group, likely attributable to the glutamic acid–facilitated nucleation and crystal maturation within the collagen matrix.

## 4 Discussion

The morphological transformation of acid-demineralized dentin served as a structural baseline for evaluating scaffold-mediated remineralization. Initial SEM images of demineralized dentin show mineral loss, widened dentinal tubules, collapse of the intertubular matrix, and reduced surface mineral density. These features are consistent with collagen degradation and mineral depletion after acid etching, as described in the study by Gandolfi et al.,.^23^ This progressive mineral deposition pattern highlights the role of nanohydroxyapatite as an effective nucleation template, enabling heterogeneous mineral growth along collagen fibrils an effect similarly demonstrated in the study by **Wang et al.** using nanobioactive glass and RGDS peptide.^24^ In PCL/nHA/Glu scaffold, SEM images showed densely packed rosette-shaped and plate-like crystallites aligned along tubule orifices by day 14. The enhanced ultrastructural organization in PCL/nHA/Glu scaffold group can be attributed to glutamic acid's biomimetic role, wherein its anionic carboxyl groups mimic the acidic domains of matrix phosphoproteins such as DMP1. This facilitates calcium ion sequestration and stabilization of ACP precursor, leading to controlled crystal nucleation and growth, **a mechanism consistent with the PILP-like remineralization pathway reported by Chen et al.,.**^25^ Consequently, the process of mineral deposition occurs in a manner that reflects the biological mechanisms of dentinogenesis. Together, these SEM observations substantiate the notion that the composition of the scaffold significantly affects not only the quantity of minerals but also the spatial accuracy and incorporation of minerals within the dentin matrix. The glutamic acid-functionalized scaffold demonstrates a superior capacity to restore microstructural characteristics resembling those of natural dentin, thereby providing a biomimetic approach for mineralization.

EDS analysis confirmed scaffold-induced mineral deposition, with increasing calcium (Ca) and phosphorus (P) signals over time in both groups. Initially Ca/P ratios remained above physiological levels, indicating immature mineral phases. Additionally, the PCL/nHA/Glu scaffold exhibited nitrogen signals corresponding to glutamic acid residue. The Ca/P ratio declined by day 28, suggesting enhanced phosphate incorporation and regulated crystal maturation**, a trend consistent with the PILP-like remineralization pathway described by Chen et al.,**.^25^ This change in ratio aligns with findings by Abdelshafi et al.,^26^ who emphasized the importance of Ca/P modulation in organized mineral growth.

The SEM results, along with the EDS findings, confirm that the PCL/nHA/Glu scaffold has excellent mineralization efficiency and biochemical compatibility. The recovery of mechanical properties in demineralized dentin reflects not only mineral presence but the quality and location of mineral integration.

To complement the ultrastructural evidence of mineral deposition, surface topography was evaluated using AFM, offering nanoscale insight into scaffold-induced architectural restoration. The pronounced surface roughness observed in demineralized dentin reflects collagen collapse and mineral depletion, findings consistent with the ultrastructural patterns reported by **Sereda et al.** using resonance-enhanced AFM-IR and nanoindentation.^27^ This irregular baseline served as a baseline for assessing scaffold-mediated remineralization dynamics.

The transient rise in surface roughness indicates early-stage cluster nucleation, consistent with the AFM observations of **Poggio et al**, who reported that initial mineral precipitation is characterized by granular surface features preceding the organization of apatite crystals.^28^ The subsequent decline in Ra, Ry, and Rz values with prolonged immersion signifies progressive mineral infiltration, surface refinement, and maturation of the deposited phase. By Day 28, stabilization of the surface roughness parameters indicates a shift from active crystal growth to organized mineral consolidation, **in agreement with Toledano et al**, who reported similar morpho-mechanical maturation during the advanced stages of dentin remineralization.^29^ The evolving roughness profile indicates that glutamic acid accelerates early mineral nucleation and directs later-stage reorganization toward a dentin-like topography. **Collectively, AFM findings confirm that while both scaffolds promote surface reconstruction, the glutamic-acid-modified variant achieves a more biomimetic and hierarchically organized restoration of the dentin surface.**

The PCL/nHA scaffold promoted a modest yet transient reinforcement, with mechanical properties peaked at 7 days, followed by a progressive decline. This trend likely reflects superficial mineral nucleation on exposed fibrils without sustained intrafibrillar integration an outcome consistent with the findings of **Bertassoni et al**,^30^ who demonstrated that complete mechanical recovery of dentin depends not merely on mineral quantity but on hierarchical mineral reconstruction and effective mineral matrix coupling.

In contrast, the PCL/nHA/Glu group demonstrated a delayed but pronounced increase in both reduced Er and HIT, particularly at Day 14, suggestive of active crystal nucleation and mineralization facilitated by glutamic acid. Its carboxylate groups chelate Ca^2+^ ions, stabilize amorphous calcium-phosphate (ACP) precursors, and promoted controlled **nucleation.**

The slight decrease in mechanical properties by Day 28 in the Glu group may reflect early remodeling or partial dissolution-reprecipitation cycles, common in biomineralization environments.^30^ Nevertheless, the sustained superiority of mechanical values compared to the PCL/nHA group confirms that glutamic acid functionalization not only enhances mineral quantity but also promotes quality and stability through matrix-bound mineral integration. These findings confirm that functional remineralization requires molecular-level reconstitution of the dentin matrix, requiring organic-inorganic synergy rather than mere ion availability. As such, glutamic acid serves as a critical mediator, recapitulating the role of native matrix phosphoproteins in dentin regeneration.

### 4.1 Limitations

The present work is limited by the absence of ultrastructural validation of intrafibrillar mineralization using TEM or SAED. In addition, in vitro immersion models cannot replicate dynamic pulpal environments, warranting in vivo confirmation.

## 5 Conclusion

The present study demonstrates that incorporation of glutamic acid into PCL/nHA nanofibrous scaffolds significantly enhances the biomimetic remineralization of demineralized dentin. Through a combination of advanced imaging and mechanical testing, it was shown that the glutamic acid-functionalized scaffold promotes earlier onset and more organized deposition of mineral phases closely resembling natural dentin architecture. Enhanced surface roughness modulation and a marked increase in mechanical properties, particularly at Day 14, underscore the scaffold's capacity to induce mineral deposition via a polymer-induced liquid precursor (PILP)-like mechanism. These findings reinforce the critical role of amino acid functionalization in mimicking the activity of non-collagenous proteins, thereby transforming passive ion delivery systems into bioactive platforms for functional dentin regeneration. Future work may explore long-term in vivo outcomes and integration with adhesive systems for translational dental applications.

## Patient's/Guardian's consent

The authors declare that this study does not include any patient-specific samples. Hence, it doesn't require anything from the patient.

## Ethical clearance

The authors declare that this study does not include any patient-specific or in vivo animal studies/samples. Hence, it doesn't require ethical clearance.

## Sources of funding

The authors declare that this study does not obtain any funding.

## Declaration of competing interest

The authors declare that they have no known competing financial interests or personal relationships that could have appeared to influence the work reported in this paper.
